# Europhile Public vs Eurosceptic Governing Elite in Hungary?

**DOI:** 10.1007/s10272-021-0959-8

**Published:** 2021-04-06

**Authors:** Borbála Göncz, György Lengyel

**Affiliations:** Institute of Sociology and Social Policy, Corvinus University of Budapest, Fővám tér 8., 1093 Budapest, Hungary

## Abstract

The threat of an epidemic narrows the scope for political competition. As Fidesz’s position within the EU has weakened significantly with the withdrawal from the European People’s Party, and the COVID-19 crisis is generating serious social tensions, the questions seem to be more open in the spring of 2022 than during the previous three elections.

Hungary was perceived to be the "good student" among EU candidate countries in the 1990s and early 2000s. However, over the past decade, the relationship between the EU and Hungary has deteriorated. Hungary and specifically its Prime Minister since 2010, Viktor Orbán, are continually criticised for violating EU values and breaking rules of EU membership. On multiple occasions, Orbán and the Fidesz regime have implemented measures that question democracy, the rule of law, freedom of the press, academic freedom and minority rights among other things. The confrontational politics were accompanied by a very symbolic domestic communication about the EU based on populist anti-EU rhetoric; most recently, this dynamic led to Fidesz quitting the European People's Party in March 2021.

Confrontations and negative domestic campaigns accompanied all stages of the EU's multidimensional crisis starting in 2008 with the global financial meltdown that lead to the eurozone crisis, followed by the 2015 migrant crisis, the subsequent challenges of illiberalism in Hungary and Poland and the most recent adversities around the COVID-19 pandemic. Different facets of the crises brought about different obstacles to European integration: supranational vs national solutions, the politicisation of the question and whether identity politics were activated (Börzel and Risse, 2018) – all of which were well reflected in the Hungarian government's anti-EU campaigns.

At the time of Hungary's accession to the EU in 2004, there was a wide societal consensus about the benefits of EU membership for the country, while the general public discourse remained technical and pragmatic. Nevertheless, the economic crisis, paired with eroding support for the government, started in 2006 in Hungary. The situation further deteriorated following the global financial and economic crisis, which led to the change in government in 2010 when Fidesz won a two-thirds majority, became a party of constitutionalising capability and managed to keep its position through the 2014 and 2018 elections. Now, on the verge of the 2022 elections, one might wonder what lies ahead.

Although a crucial and oft-cited element of the Fidesz government is that it represents and defends the interests of Hungarian people, it appears that the Hungarian people are not that hostile toward the EU as their repeatedly re-elected government. It seems that, in line with the phenomenon of rising public Euroscepticism all over Europe, Hungarian public support tended to decrease until 2012 but has been on the rise since then despite the negative impact that one might expect due to the anti-EU government rhetoric. The fact that general public opinion does not reflect the changed elite discourse may be due to pragmatic attitudes towards the EU valuing the economic benefits that it represents (Lengyel and Göncz, 2010). An alternative explanation is that there is an increasing satisfaction with Hungary's economic and political performance, which is reflected in the attitudes of Hungarians towards the European integration project. Yet another possible explanation for the prevailing diffuse support for the EU in Hungary is based on emotional attachment or past identification; however, despite being rather strong in Hungary, this element did not have a significant impact on support for the EU (Lengyel and Göncz, 2010). In the following, attitudes of the general public as well as those of Hungarian political elites are considered.

## Support for the European integration project in Hungary

The legitimacy and the stability of a political system partly comes from the general support for it. System theorists distinguish between *specific support* based on the perception of the performance of a system and *diffuse support* representing a reservoir of positive attitudes towards a system that make people accept non-favourable outputs (Easton, 1965; Harteveld et al., 2013; Ringlerova, 2015). Scholarly work supporting the European integration project mirrors this distinction as the main explanatory models elaborated so far revolve around utilitarian logic and explanations based on identification that could

* This article was written in the frame of the MEDIATIZED EU project funded by the European Union's Horizon 2020 research and innovation programme (Grant nr. 101004534).

**Borbála Göncz**, Corvinus University of Budapest, Hungary.

**György Lengyel**, Corvinus University of Budapest, Hungary.

be considered manifestations of specific and diffuse support respectively. In utilitarian logic, attitudes are defined by a rational evaluation of the EU's advantages and disadvantages at the individual or at the country level (e.g. Gabel, 1998; Brinegar and Jolly, 2005; McLaren, 2006), or explanations focused on the perception of the functioning of the EU, i.e. how efficient one finds European institutions (e.g. Opp, 2005). Starting from the 2000s, identity-based explanations appear to challenge the utilitarian ones, with a focus on the impact of affective or emotional attachment to Europe based on perceptions of the European integration process (e.g. Duchesne and Frognier, 1995; Hooghe and Marks, 2005; Opp, 2005; Bruter, 2005; Risse, 2010). According to studies focusing on concepts such as identity, belonging to a group or loyalty, identity-based explanations have an increased relevance among the general public due to a lower level of cognitive capacities, knowledge of or interest in the issue (Hooghe and Marks, 2009). Nevertheless, the concept of European identity is very much contested in the academic community in terms of its content, plausibility or possible measurement (Favell, 2005).

Similarly, those arguing that the European integration process is too complicated or remote for ordinary people to understand or be interested in should remember that people might rely on familiar proxies in order to form an opinion. The domestic political arena and the perception of national political and economic performance is thus important as they create the partys' stance and messaging (e.g. Gabel, 1998; Anderson, 1998; Carrubba, 2001; Steenbergen et al., 2007; Hooghe and Marks, 2009).

## Attitudes of the general public

During the 1990s and early 2000s, there was a wide societal consensus in Hungary about its accession to the EU. This period was characterised by a broad public discourse about the "return" of the country to Europe. However, general positive attitudes were paired with widespread ignorance. About one-third of the population had a positive perception of the EU's objectives and activities, another one-third held a neutral opinion, and the remaining third was divided between those without an opinion (21%-34%) and those opposed to the EU (6%-11%). Support for the country's EU membership reached its peak in 2002 and began a steady decline thereafter (Lengyel and Göncz, 2010). Nevertheless, after a successful referendum on the accession in 2003 with 84% in favour (albeit with a turnover of 46%), general support was still above the EU average at the time of the accession in 2004.

Data show that this decreasing tendency prevailed up until 2012, but since then the share of positive perceptions follows an increasing tendency besides a steady share of

Figure 1

**Image of the EU in Hungary**

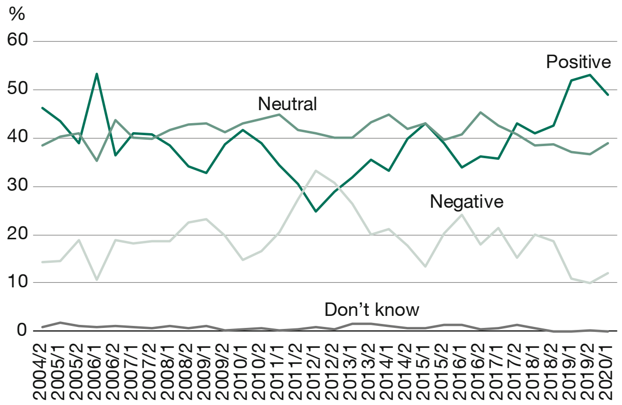

Notes: The wording of the question is: "In general, does the European Union conjure up for you a very positive, fairly positive, neutral, fairly negative or very negative image?". Very/fairly positive and fairly/very negative are regrouped. There are several survey questions used to assess the general attitudes towards the EU and the perception of a country's EU membership. The current question about the image of the EU is able to grasp general attitudes and has the advantage of being available for the entire period considered. Furthermore, despite the variety of questions and the specific advantages and limitations of each, according to previous experiences these questions reflect the same reality and show similar tendencies.

Source: Eurobarometer.

neutral opinions (38%-45%). Figure 1 shows that 46% of Hungarians had a positive image of the EU in 2004 as opposed to 24% in 2012; most recently, positive perceptions climbed up to 49% in 2020. Similarly, 14% had a negative perception of the EU in 2004. This sentiment peaked in 2012 when it went up to around one-third of the population and then decreased by 2020 to 12%. As we can see, while Hungary was among the most Eurosceptic countries in 2012, it was one of the most supportive ones by 2020.

Previous studies seem to confirm that the elections might have a positive impact on the perceptions of the EU, opinions generally being more positive in the year of elections – and this was the case in 2002 and 2006 (Lengyel and Göncz, 2010). However, with the change in government in 2010, this relation appears to be more blurred, perhaps due to the intense negative campaign against the EU after Fidesz took power in 2010. Despite the constant presence of negative discourses and campaigns from the governing elite since 2010, positive public perceptions of the EU increased. This raises several questions about the mechanisms behind public opinions. While it has been suggested that utilitarianism had a more individual character in Central and Eastern European countries (McLaren, 2006), utilitarian evaluations might have become even stronger after the financial and subsequent economic crises (Hobolt and Wratil, 2015), which may be the reason why the Hungarian

Figure 2
**Attachment to the EU in Hungary**

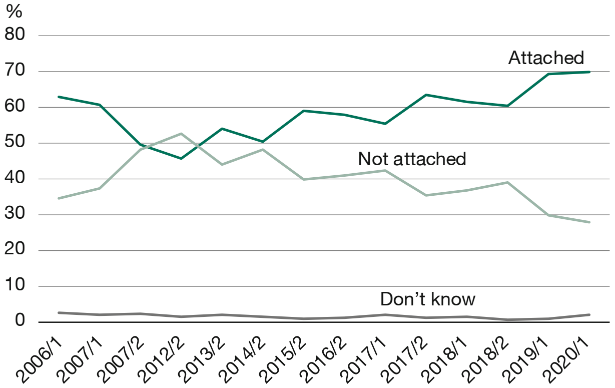

Notes: The question is: "People may feel different degrees of attachment to their town or village, to their region, to their country or to Europe. Please tell me how attached you feel to the European Union: Very attached/ Fairly attached/ Not very attached/ Not at all attached/ Don't know". Very/fairly attached and Not very/not at all attached are shown together.

Source: Eurobarometer.

government's negative messaging did not make a great impact.

Besides general attitudes, it is worthwhile to look at emotional attachment as a potential measure of diffuse support, e.g. in the early 2000s, Hungarians seemed to be strongly attached to Europe, yet perception of the country's EU membership was less enthusiastic (Lengyel and Göncz, 2010). Comparing Figures 1 and 2 reveals that attachment is higher than the positive evaluation of the EU. It is equally important, nevertheless, that these two measures follow similar trends. While in 2006, 63% of the population claimed to be very or somewhat attached to the EU, by 2012 this figure had decreased to 46% and increased again to 70% by 2020, putting Hungary in the ranks of the most Europhile countries. Similar to the previous survey of general perceptions of the EU, attachment was at its lowest in 2012 but increased again by 2020. Nevertheless, although Hungarians are quite attached to the European project, identification has not proved to be a significant determinant of the support for the EU in the past (Lengyel and Göncz, 2010).

Hungarian public opinion about the European integration process is not homogeneous either. Similar to other countries, education and urban environment seem to be a catalyst of the support. Furthermore, the embeddedness of the subject in the domestic political arena is very important.

## Perception of the political elite

After the post-socialist transformation of Hungary, there was an elite consensus about the Euro-Atlantic orientation. A large consensus accompanied the whole negotiation process and accession of the country to the EU – only Fidesz, then the opposition party, expressed reservations. Nevertheless, the Hungarian Parliament voted unanimously for accession. The 2010 elections, however, mirrored citizens' dissatisfaction with the political elite and led to significant changes in party structures and the national parliament. Fidesz won the elections while a right-wing extremist party Jobbik got into the parliament, and parties of the transition like the liberal SZDSZ and the conservative MDF disappeared. The change of the political and economic context resulted in a significant shift in public discourse about the EU as well as the EU increasingly appearing in a negative light based on symbolic messages.

Previous studies point to the fact that either due to their higher exposure to EU matters or their status, European political elites hold more positive opinions about the EU than the general public. Hungary was not an exception to these trends up until the change in governance in 2010. However, by 2014 the views of Hungarian parlamentarians changed to resemble those of the general public. In a political and economic context characterised by rising public Euroscepticism and an increasing gap between the public and their elites after the financial and economic crisis, Hungary showed very different tendencies, as seen in Figure 3a. The gap first increased and then strongly decreased by 2014 (Vogel and Göncz, 2018).

Although previous studies suggest that the polarisation of political elites in political issues should be higher than the polarisation of the general public (McAllister, 1991), in terms of variation of opinions about European integration matters, elites are generally less polarised than the general public, but Hungary was an exception in 2014 (Vogel and Göncz, 2018). Besides these general tendencies, preferences for supranational institutional design prevailed in 2007 and 2009, while Hungarian parliamentarians became rather state-centred by 2014 adopting a more intergovernmental idea of the European construct. Representatives of the dominant governing party, Fidesz, believed that European integration had gone too far, that the EU endangered Hungarian culture and generally trusted EU institutions less than the small oppositional groups of socialists and greens. In this respect, the dominant governing party differed sharply even from its almost invisible pro-EU Christian Democrat satellite. Nevertheless, even after the change in government, in 2014 the majority of parliamentar-

Figure 3
**Perception of the European integration project and attachment to Europe in Hungary among the general public and the political elites**

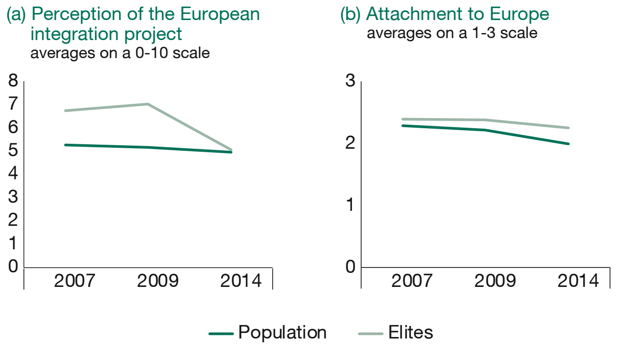

Notes: (a) The wording of the questions measuring the perceptions of the European integration project is: INTUNE/ENEC: "Some say European unification should be strengthened. Others say it already has gone too far. What is your opinion? Please indicate your views using a 10-point-scale. On this scale, "0" means unification 'has already gone too far' and "10" means it 'should be strengthened'". EES: "Some say European unification should be pushed further. Others say it already has gone too far. What is your opinion? Please indicate your views using a scale from 0 to 10, where "0" means 'unification has already gone too far' and "10" means 'it should be pushed further'". (b) The wording of the questions measuring attachment is: INTUNE/ENEC: "People feel different degrees of attachment to their region, to their country and to Europe. What about you? Are you very attached, somewhat attached, not very attached or not at all attached". EES: "For each of the following statements, please tell me to what extent it corresponds or not to your attitude or opinion. You feel attached to Europe". Answers: Yes, totally/ Yes, somewhat/ No, not really/ No, not at all. Values have been recoded so as to have the averages on a 0-3 scale, where 3 stands for "very attached"/ "Yes, totally" and 0 stands for "not attached at all"/ "No, not at all".

Source: INTUNE (2007, 2009) and ENEC (2014) projects and the 2014 Voter Study of the European Election Study for the general public in 2014 (Vogel and Göncz, 2018).

ians still felt that the country has benefited from the EU membership (Göncz and Lengyel, 2016).

Attitudes in terms of attachment to Europe, on the other hand, proved to be more stable with a less notable difference between parliamentarians and the general public as well as less change over time (see Figure 3b).

## What might the future hold?

The fact that the negative symbolic governmental messages and campaigns in Hungary did not have a deteriorating effect on the general support for the European integration process since 2012 may eventually be due to utilitarian logic, which might have prevented a significant impact of symbolic messages and could represents the relevant frame of reference when evaluating the European integration process (McLaren, 2006; Lengyel and Göncz, 2010).

Figure 4
**Trust in national government and the EU in Hungary and in the European Union**
Percentage of respondents who "tend to trust" their national government and the EU

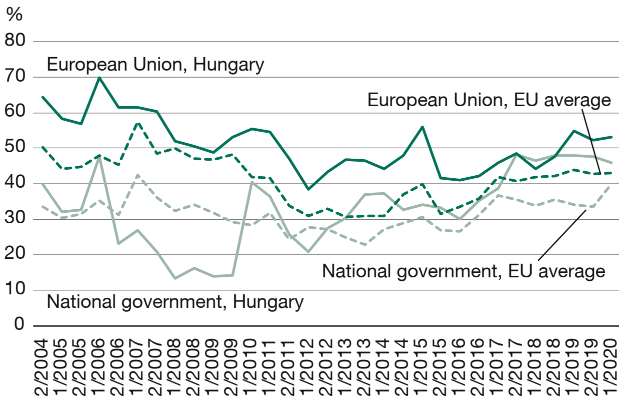

Source: Eurobarometer.

It seems that the Hungarian general public is engaged with the European project even if the government – supported by the same public – is very critical about it. Taking into account the important embeddedness of the subject in the domestic political arena, this might seem a contradiction.

Looking at trust in institutions, a possible measure of perception of the performance of a system and a measure of its legitimacy, one can see that trust in the EU is somewhat higher than trust in the national government in general; however, this is even more pronounced in Hungary, at least in the period leading to the change in government in 2010 (see Figure 4). This gap closed, however, in the period 2017-2018, when trust in the two institutions was even. Trust in government has rather stagnated around 47% since then, while trust in the EU increased somewhat. Looking at the period before 2010, it seems that trust in the government and trust in the EU follow similar tendencies, with trust a little higher around the elections before 2010 (spring 2006, spring 2010). This does not apply to the elections after 2010 when no change in governance occurred. Similar to the tendencies presented earlier, 2012 is a negative peak in terms of trust in institutions. On the other hand, since that low point, institutional trust follows a rather increasing tendency, especially with regards to the Hungarian government with opinions stagnating in the last two waves. This eventually points in the direction of a sociotropic utilitarian or cueing rationality logic, which states that the increasingly positive perception of the EU reflects an increasing satisfaction with Hungary's economic and political performance – represented by a higher trust in the government.

This raises a question about the outcome of the upcoming Hungarian elections in 2022 as during all elections since 2010: Can the Fidesz government be voted out of office? Despite high hopes from the united opposition, the level of public trust in the actual government suggests caution.

It seems that the change in the political elites' structure and rhetoric has not affected public attitudes towards the EU significantly. Although previous research confirmed the effect of media news on attitudes in questions related to the European integration project (Bruter, 2003), these do not seem to be reflected in the Hungarian case. While anti-EU rhetoric started around 2010, Euroscepticism started to increase earlier and seems to be decreasing from 2012, although there were no changes in communication or political preferences of the government. Several possible explanations arise. The most probable one is the suggestion that public opinion about the EU is very much utilitarian and pragmatic in Hungary and, despite government communications, EU membership still holds advantages for the country or at the individual level. Another possible explanation is that Hungarians are strongly attached to European values, which indicates diffuse support and means that their attitudes towards the EU are less prone to change. This explanation is somewhat disproved by previous findings that identification is not a key driver of general support in Hungary (Lengyel and Göncz, 2010). A third possible explanation is that Hungarians are satisfied with the economic and political performance of the country under the Fidesz regime, proved by their increasing trust in their government, and, as a "cue", they extrapolate their satisfaction to the supranational level. In any case, trust in the Hungarian government seems to have stagnated since 2017, while trust in the EU is more volatile; still, according to the most recent data, it exceeds the trust in the Hungarian government.

All in all, the question of what drives the public attitudes in this case needs further research. A very practical implication of what has been described is the future of Hungary in the European Union: How much can the perception of a government be separated from the actual people (from the point of view of the EU)? How long will this gap between the governing elite's discourse and public opinion be maintained? What will be the implications of the outcome of the next elections of this question? And could the question of EU membership lead to an eventual change in governance?

It depends on how successfully Fidesz pursues blame-game tactics concerning the EU, diverting attention from government failures and strengthening the clientele, and how functional the opposition coalition will be. The threat of an epidemic narrows the scope for political competition. As Fidesz's position within the EU has weakened significantly with the withdrawal from the European People's Party, and the COVID-19 crisis is generating serious social tensions, the questions seem to be more open in the spring of 2022 than during the previous three elections.
